# The Association between Hypertensive Disorders of Pregnancy and Peripartum Cardiomyopathy

**DOI:** 10.7759/cureus.5867

**Published:** 2019-10-08

**Authors:** Kiran F Rana, Aisha Saeed, Sohaib A Shamim, Muhammad Ali Tariq, Bilal Haider Malik

**Affiliations:** 1 Family Medicine, California Institute of Behavioral Neurosciences and Psychology, Fairfield, USA; 2 Neurology, California Institute of Behavioral Neurosciences and Psychology, Fairfield, USA; 3 Internal Medicine, California Institute of Behavioral Neurosciences and Psychology, Fairfield, USA

**Keywords:** hypertensive disorders of pregnancy, peripartum cardiomyopathy, preeclampsia, association of hypertensive disorders of pregnancy, cardiomyopathy, hypertension during pregnancy, heart failure during pregnancy and postpartum, peripartum mortality, idiopathic cardiomyopathy during pregnancy, cardiovascular mortality during and after pregnancy

## Abstract

Peripartum cardiomyopathy (PPCM) is a diagnosis of exclusion and a heterogeneous disorder that presents during the last month of pregnancy or the first five months postpartum. It is a rare but potentially life-threatening illness. A lot of work has been done trying to discover the causes of this condition, and several risk factors have been identified, including hypertension during pregnancy (HDP), ethnicity, advanced age, and multiple gestations. HDP affects 40% of cases of PPCM, and the strength of the association increases with increasing severity of hypertension. Among PPCM patients, there is a 1.5 times higher prevalence of HDP and a four-fold higher prevalence of preeclampsia (PE). Besides, the risk of PPCM markedly increases among women with HDP (5-21 times) compared with normotensive women. The experimental work done in animal models has provided support for the angiogenic-imbalance theory proposed regarding the association between these two conditions. The presence of the same risk factors also supports the prevalence of the coexistence of PE and PPCM. During the last part of gestation, the placenta secretes more anti-angiogenic factors, which leads to the development of both PE and PPCM. However, not all patients with HDP develop PPCM. In fact, most PPCM patients do not show any signs of HDP. Further work in these patients elucidated that there is an underlying susceptibility in some women that predisposes them to develop this condition and results in a worse prognosis as compared with those PPCM patients who have HDP. Better provision of care, genetic variations, and association with HDP have been cited as some of the factors affecting prognosis. HDP has also been found to increase the risk of other forms of cardiomyopathies in the future. A lot of work still needs to be done to uncover all the pathologic mechanisms and genetic variations involved in this disorder. More intensive and focussed research may help in developing new therapies to better manage this condition and address all of its complications.

## Introduction and background

The connection between postpartum heart failure and hypertension was first described in 1938 by Hull and Hidden. They observed that postpartum heart disease was associated with hypertension and occurred twice as often in women with hypertension during pregnancy than in the control population [1]. Peripartum cardiomyopathy (PPCM) is a form of idiopathic systolic heart failure that develops during the last month of pregnancy or the first five months postpartum. The condition is characterized by a left ventricular ejection fraction (LVEF) of <45% and/or fractional shortening of <30%. There is no apparent link between PPCM and heart failure or a history of heart disease before pregnancy [2].

According to the recent data, PPCM occurs in 1/1,000-1/4,000 pregnancies, more commonly in women of African ancestry [3,4]. It may be found in up to 1% of all pregnancies in countries like Haiti and Nigeria [5,6]. The perinatal outcomes may be dismal in terms of maternal and infant mortality, accounting for 5% of cardiac transplants in US women [7]. Only 25% of PPCM patients in developing countries survive up to five years, with associated infant mortality of 50-75% [8]. In the US, there is almost a 6% mortality rate for non-Hispanic white women, whereas it is elevated four times in women of African origin [9]. Although the underlying pathophysiological mechanism of PPCM is not known, this data highlights the increased prevalence of PPCM in certain races. Other commonly observed predisposing factors include advanced age, multiple gestations, and hypertensive disorders, including but not limited to gestational hypertension and preeclampsia (PE) [10].

PE has more frequently been associated with PPCM of late [11]. However, some studies have failed to find a link between these two conditions. With our study, we aim to explore the association between hypertensive disorders of pregnancy (HDP) and PPCM by examining all the available data.

## Review

Methods

A preliminary search was done using PubMed for this review article. We performed the search using several keywords and Medical Subject Headings (MeSH) keywords, and the tables containing those words and the related number of articles are included here (Tables 1 and 2).

**Table 1:** PubMed search with keywords

Keywords	Database	No. of results
Hypertension	PubMed	382,638
Cardiomyopathy	PubMed	89,447
Preeclampsia	PubMed	35,247
Hypertensive disorders of pregnancy	PubMed	3,881
Peripartum cardiomyopathy	PubMed	1,225
Hypertensive heart failure of pregnancy	PubMed	173

**Table 2:** PubMed search using MeSH keywords MeSH: Medical Subject Headings

MeSH Keywords	Database	No. of results
Cardiomyopathy	PubMed	104
Preeclampsia	PubMed	4
Hypertension during pregnancy	PubMed	3

The largest number of articles was found when PubMed search was carried out using "hypertension" as the keyword. The MeSH keyword "cardiomyopathy’" also returned the largest number of articles. A more focussed search on our current topic, "hypertensive disorders of pregnancy and peripartum cardiomyopathy", brought up 40 articles. The second search using "preeclampsia and peripartum cardiomyopathy" returned 128 articles. We excluded studies published in other languages by applying the "English language only" filter. The data from both these topics were combined to cover the available information relevant to our study. Since it was a traditional review, we did not use Prisma or Amstar guidelines. We ensured that all our work adhered to the ethical principles and guidelines outlined for research studies. There was no restriction regarding the time of publication or the type of study for the articles selected for review. Among the included studies, 141 were done on humans, and 27 were carried out in animals.

HDP occurs in 6-8% of pregnancies and is characterized by chronic hypertension, PE, PE superimposed on chronic hypertension, gestational hypertension, and eclampsia. Gestational hypertension is defined as high blood pressure (BP) (>140/90 mmHg) without proteinuria (>0.3 g/24 hours). In PE, there is proteinuria along with mild/moderate hypertension. Severe preeclampsia includes high BP with proteinuria and may present with one or more of the following features: 1) severe hypertension (≥160/110mmHg); 2) significant proteinuria (≥5 g/24hours or ≥3+); or 3) signs of organ failure [12].

Results

A total of 165 articles were identified as potentially relevant and were reviewed for our study. A list of some of the high-yield articles along with study characteristics is included here (Table 3).

**Table 3:** Studies showing a strong relationship between HDP and PPCM HDP: hypertensive disorders of pregnancy; PPCM: peripartum cardiomyopathy; PE: preeclampsia; sFlt-1: soluble fms-like tyrosine kinase-1

Authors	No. of patients	Study type	Year	Country	Study objective	Findings
Behrens et al. [13]	126	Cohort	2019	Denmark	The authors examined the relationship between HDP and PPCM by choosing a cohort of Danish patients with ≥1 live or stillbirth from 1978 to 2012.	Despite finding 70% of PPCM patients to have normal blood pressure, HDP was significantly associated with an elevated risk of PPCM, which was proportional to the degree of hypertension.
Bello et al. [14]	979	Meta-analysis	2017	USA	The study systematically reviewed available studies on PPCM to calculate the incidence of PE and performed secondary analyses using hypertensive disorders, multiparity, and multiple gestations.	The combined incidence of 22% was more than four times the 5% average global incidence of PE. There was an increased occurrence of multiple gestations and other HDPs.
Kamiya et al. [15]	102	Retrospective cohort	2011	Japan	This study was carried out to observe the characteristics of PPCM in Asian (Japanese) women and to observe the difference in outcomes for patients with and without HDP.	There is a favorable outcome for patients with PPCM who are complicated with HDP. They show rapid recovery if the patient survives. To decrease cardiovascular mortality, patients with HDP should be closely monitored for signs and symptoms of heart failure.
Ersboll et al. [16]	219	Cohort	2017	Denmark	This study aimed to measure the incidence and looked at the outcomes of PPCM in Danish patients from 2005 to 2014.	The incidence of PPCM ranged from 1-10 per 149 deliveries. The women with PPCM who had HDP showed greater improvement in cardiac status.
Mebazaa et al. [17]	178	Cohort	2017	South Africa, France	Changes in the angiogenic balance are seen in patients with PPCM.	Levels of anti-angiogenic factor, soluble fms-like tyrosine kinase 1 (sFlt-1), are increased in patients with PPCM.
Kathryn et al. [18]	39	Retrospective cohort	2013	USA	The study looked for the impact of PE on clinical or left ventricular functional outcomes in women with PPCM.	There is increased morbidity and mortality in patients with PPCM if they have concurrent PE.
Kamiya et al. [19]	417	Review article	2016	Japan	The study evaluated the genetic factors associated with PPCM both in patients with and without HDP.	There are common genetic elements seen both in patients with PPCM and those with dilated cardiomyopathy (DCM).15% of PPCM patients had genetic variations found in dilated cardiomyopathy and had lower recovery rates.

To explore the association between HDP and PPCM, a cohort study was done in Denmark by Behrens et al. It identified 126 women with PPCM, 39 of whom developed HDP whereas 87 were normotensive [13]. Most cases (82% vs. 81.6%) were diagnosed either during the final month of pregnancy or the first month of postpartum, both in women with or without HDP. HDP was found to be strongly associated with PPCM, and the magnitude of this association was proportional to the degree of hypertension. Women who were more than 35 years old had multiple gestations had a higher incidence of PPCM, whereas increasing parity was not found to be a significant risk factor for PPCM.

Bello et al. found that the overall prevalence of PE among patients with PPCM ranged from 0-78% in the included studies [14]. They found the average prevalence to be 22%, which was more than four times the average worldwide prevalence of 3-5% [probability value (p): <0.001]. It was attributed to significant heterogeneity among the included studies. Upon further analysis of the rates of PE by country, it was found that the studies done in the US had a prevalence of 24% [95%; confidence interval (CI): 17-32], which was higher than in other parts of the world, where the estimated prevalence was 17% (95%; CI: 8-26). The studies from other countries had significantly decreased heterogeneity. Other patient characteristics like the presence of any hypertensive disorder (preeclamptic or not), multiparity, and multiple gestations were also assessed for association with PPCM. The results showed an increased prevalence of both multiple gestations and hypertensive disorders in women affected by PPCM compared to baseline rates in the general population. The range for the presence of any hypertensive disorders was 2-83%, with an average of 37%, which was substantially more than an estimate of 22-27%. The incidence of multiple gestations ranged from 0-38%, with an average rate of 9%, which was again more than the estimated average prevalence of 3%. Multiparity was seen in two-thirds of the patients with PPCM. The disorder is more common in females of African descent, indicating that the association between PE and PPCM may be different in this group of women [3,9]. However, no correlation was observed between the incidence of PE and African heritage in PPCM patients (correlation coefficient: −0.35; p: 0.4).

In another study by Kamiya et al., which looked at the characteristics of PPCM, 42 patients were found to be complicated with HDP (HD+ group), and 60 patients were without the complication (HD- group) [15]. There was a higher incidence of PPCM in relatively older women, more commonly in the (HD+) group and was >10-fold elevated in 35-39-year-old women than in 20-24-year-old women in the same group. However, it was only three times higher in the HD- group. The two groups exhibited similar cardiac function parameters and brain natriuretic peptide (BNP) levels at diagnosis.

In the article by Ersbøll et al., almost half of the women with PPCM had HDP. These women had higher baseline LVEF as compared to those without HDP, were more likely to be primiparous, had an earlier diagnosis, and stayed in the hospital for a shorter duration [16]. They also required less cardiovascular support and showed better outcomes with increased rates of complete recovery. Their mean LVEF at a one-year follow-up was higher compared with patients without HDP [55 (±7) vs. 50 (±9); p: 0.01). The complete recovery numbers reported in this study were not statistically significant (p: 0.129), presumably due to a small sample size. However, similar reports of better outcomes in PPCM patients with HDP have been published in previous studies [15].

Discussion

**Figure 1:**
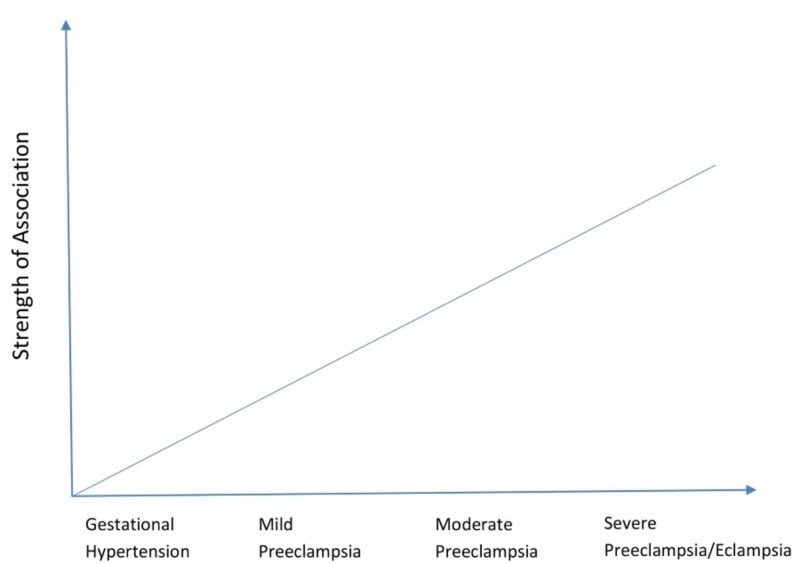
The relationship between HDP and PPCM HDP: hypertensive disorders of pregnancy; PPCM: peripartum cardiomyopathy

Many studies have been done to find out if HDP, especially PE, and PPCM are two uniquely different clinical disorders or if they are manifestations of similar pathophysiological processes. It is still the subject of an ongoing debate. However, a strong association between HDP and PPCM has been found in studies specifically looking for such an association. Bello et al. found 1.5 times higher rates of HDP and a four-fold increase in the incidence of PE in patients with PPCM compared with the control group [14]. Much of the previous work shows that PPCM risk might depend on HDP severity [11,20,21]. Several studies have been carried out at different subpopulations in many countries that sheds light on the various common and different characteristics observed for the two phenomena. The reported incidence of HDP among PPCM patients ranges from 2-68%.In the most recent study by Behrens et al., a cohort of >2 million pregnant females across Denmark revealed that the risk of PPCM in women with HDP was 5-21 times higher than in normotensive women, and the risk increased with increasing severity of hypertension [13] (Figure 1).

Physiological Processes Involved

PE reversibly affects many organs including the liver, kidney, brain, and the hematological system. However, the heart is usually spared in PE and echocardiography merely shows increased afterload due to hypertension and a decreased preload that can be improved with hydration [22,23].

Recent studies have shown that deterioration in diastolic function precedes systolic dysfunction in PPCM, which is similar to the end-stage dilated phase of hypertensive cardiomyopathy. There was also a weak positive correlation between the duration of PE and LVEF at diagnosis, which indicates that hypertension exacerbates PPCM in the initial phase [24]. Up to 80% of preeclamptic women tend to develop some degree of myocardial adaptive changes, and around 50% exhibit left ventricular diastolic dysfunction. Only 20% of women who have severe PE develops significant cardiac problems characterized by left ventricular hypertrophy and systolic dysfunction [25]. This cardiac dysfunction lasts for at least 12 months, even when adequate treatment for hypertension is provided [26].

Pathophysiological Processes for the Two Phenomena

Several pathophysiological mechanisms have been proposed for the development of PPCM, including pregnancy-induced cardiac overload consisting of hypervolemia, elevated heart rate, and thrombophilia. But recent evidence suggests that some PPCM patients may have the same presentations as familial dilated cardiomyopathy (DCM) since HDP usually presents with LV diastolic dysfunction and may not lead to LV systolic dysfunction found in PPCM [2,27-29].

PE is generally considered to be a vascular disease that appears during the later part of pregnancy when the placenta secretes increasing levels of antiangiogenic factors into maternal blood [30]. These factors are found to be strongly associated with PE [31]. One of the most common antiangiogenic factor, soluble vascular endothelial growth factor receptor 1 (sVEGFR1), attaches to and inactivates circulating vascular endothelial growth factor (VEGF), which at high levels disturbs homeostasis in many vessels, including glomerulus, thereby leading to proteinuria and high BP of PE [32,33]. Recent studies from two mice models also found that PPCM is a disease involving angiogenic imbalance [34,35]. In one model, the deficiency of peroxisome proliferator-activated receptor-gamma coactivator 1-alpha (PGC1α) resulted in more serious PPCM and DCM even in those without pregnancy. Moreover, pro-angiogenic therapy with recombinant VEGF ameliorated PPCM. This further emphasizes that PPCM is a vascular disorder and the shared pathology of PE and PPCM may be the potential explanation for the strong link between the two conditions [14,34].

Molecular/Biochemical Basis for the Association

The timing of secretion of sVEGFR1 and other anti-angiogenic factors explains how it plays a crucial role in triggering PPCM, which tends to occur towards the end of pregnancy instead of the second trimester when there is the maximum hemodynamic load for the heart [36]. Besides, there is a frequently higher incidence of PPCM in women with twin pregnancies, which is due to larger placentas secreting more sVEGFR1 into the maternal blood [37]. Similarly, severe PE is more strongly associated with PPCM since it often leads to higher levels of anti-angiogenic factors [34]. This clinical and experimental data gives rise to a two-step theory as the cause of PPCM. The first step is increased susceptibility in certain women due to unknown factors, which may be a genetic or familial predisposition to develop PPCM. More research needs to be done to explore these unidentified contributory factors. The second step is an elevated level of cardiotoxic sVEGFR1 and other antiangiogenic factors during late-gestation. PE and twin pregnancies exaggerate the second step, giving rise to robust manifestations of PPCM [14].

Risk of other Cardiovascular Disorders in Patients with HDP

HDP also being recognized as a factor that increases the risk of developing cardiovascular disease in the future. A history of PE increases the risk >2-times [33], which is similar to the risk posed by other major risk factors like hyperlipidemia and diabetes. Therefore, the American Heart Association now recommends checking for a history of PE to assess the overall cardiovascular risk in all women [38]. Whether this increased risk is due to the pathophysiology of PE or common risk factors remains to be examined.

Factors Affecting Prognosis in PPCM Patients

Regarding the prognosis of PPCM, some of the parameters that affect the outcome are the presence of HDP, LVEF at diagnosis, left ventricular diameter (LVD), left ventricular thrombus, and ethnicity [17,39]. Kamiya et al. reported that despite having similar cardiac indices at diagnosis and discharge, those with HDP had a shorter hospital stay and showed greater improvement in LVDs, %FS (percentage fractional shortening), and LVEF at the last follow-up [15]. Ntusi and Mayosi also revealed that PPCM patients with HDP had good recovery of left ventricular function at six months [40]. These observations contradict the association between PE and PPCM but can be explained by the provision of differential/better care in the group with HDP. Another important finding from the study by Ersboll et al. was that the patients treated with labetalol had better outcomes during follow-up at one year. However, the study did not provide details on the number of labetalol-treated patients who did or did not have HDP [16]. This leads to an important question: would the use of medications for HDP be justified in PPCM patients without HDP to improve the outcomes? Answering this question may not only add a mortality benefit but also uncover the pathological mechanisms involved in PPCM. A new clinical trial can be designed to observe the effect of labetalol and other related therapies in PPCM patients with or without HDP. Labetalol is used as the first-line drug for treating HDP in Denmark and has been found to increase VEGF and reduce sFLT1 in IV studies [41].

Another reason cited for the poor prognosis in some patients without HDP is the presence of a positive family history of DCM, which is found in as many as 10% of the patients [42,43]. Through genetic sequencing of PPCM patients for 43 genes and their variants associated with DCM, it was found that the prevalence of certain truncating variants was markedly elevated than the reference population but closely matched patients who had DCM [44]. Significantly, 67% of the discovered truncating variants were in the TTN gene. TTN codes for a protein, titin, which is one of the three basic filaments of the cardiac sarcomere, and its truncated variants have been identified in 20% of DCM cases. In a study done on PPCM patients, the existence of TTN truncating variants was associated with lower LVEF at a one-year follow-up [44]. Another interesting finding from DCM genome sequencing in PPCM patients was that patients with TTN variants had a very low prevalence of HDP (1/11; 9%), compared to those without the truncating variants who had a much higher prevalence of HDP (35/68, 51%, P: .009) [45]. One plausible explanation for the better outcome is linked to the common pathological mechanism of the two conditions and is supported by the fact that PE tends to improve rapidly after delivery, leading to the recovery of LVEF and the decline in anti-angiogenic factors.

Many authors believe PPCM with HDP to be a subset of PPCM and, due to diagnostic difficulties in pregnant patients presenting with edema and dyspnea, it is beneficial to check serum BNP levels as well as chest x-rays to deliver the right treatment at the appropriate time [15]. This can decrease the mortality associated with diagnostic delay and prevent worse outcomes such as death or heart transplantation [46].

Future Areas to Explore

Despite the strong association between HDP and PPCM, most cases of PPCM (69%) occur in healthy normotensive pregnant females [13]. Certain susceptible women are unable to handle the hemodynamic stresses of pregnancy. A recent study showed that 18-28% of healthy nulliparous women had some degree of cardiac dysfunction at term [47].

The underlying susceptibility often appears to be more important than the association between HDP and PPCM. Most studies have failed to provide a causative link between HDP and PPCM. However, they suggest that both conditions have common risk factors involved in the pathophysiology, which accounts for the coexistence of the two conditions in many cases [15]. Higher levels of anti-angiogenic factors (e.g., sFLT1) are found to be involved in causing HDP. The same anti-angiogenic factor (sFLT1), as well as prolactin cleavage products, have also been observed to cause PPCM, but these are not the only factors involved [34,35,48]. A more thorough study needs to be carried out to look for other anti-angiogenic precursors so that the therapy could be targeted to better manage these two conditions. It still does not explain the fact that most cases of PPCM are in women without HDP, which signifies some underlying predisposition perhaps related to the inheritance of particular genes. So far, all we know is that in the presence of truncated variants of TTN in women already prone to develop DCM, the additional hemodynamic load of pregnancy may result in an overt presentation of PPCM [44]. What other genes or their variants could be associated with PPCM remains to be discovered.

The focus of most research work to date has been on PE and PPCM as shown in the meta-analysis performed by Bello et al. examining the relationship between the two [14]. However, there is a growing need to design and perform more studies looking at the etiology and treatment of various pathologies involved in the causation of PPCM, especially in patients with HDP. This can provide concrete evidence for the underlying mechanism as well as the association between the two conditions.

Limitations

Although this study is useful in terms of compiling the information available on the topic of association of HDP and PPCM, we could not answer some questions that arose during our work, which emphasizes the need to designing more research projects. Some of the studies had excluded patients based on various reasons, which, if included, could uncover further dimensions of the pathophysiology. Many patients in the earlier studies were not included because of the diagnostic difficulties. For instance, pulmonary edema due to PPCM was often confused as perpetrated by PE. There was only one meta-analysis available that looked at the association between PE and PPCM, but no meta-analysis examining the relationship between HDP and PPCM existed in the pool. There were not many animal studies available on the subject. However, some of the most valuable findings regarding the pathophysiology of PPCM emanated from experiments on animal models. There is a dearth of clinical trials comparing the treatment efficacy and factors affecting long-term prognosis in PPCM patients with or without HDP. Although many authors agree on the angiogenic imbalance as the underlying pathological mechanism in PPCM patients with HDP, no therapeutic trials have been carried out in humans that used pro-angiogenic therapy as the potential cure for the condition. Further work needs to be done looking at the genetic factors, which may be involved in causing PPCM in various subsets of patients, e.g., those having HDP, family history of DCM, or other cardiomyopathies.

## Conclusions

Our study aimed to explore the association between HDP and PPCM through a careful review. All the gathered evidence shows that a strong relationship does exist between HDP and PPCM. The strength of the association is directly proportional to the severity of hypertension. Clinically, approximately 40% of PPCM patients are found to have HDP. Both HDP and PPCM are vascular disorders that share the same risk factors and a common pathological mechanism related to angiogenic imbalance. That is why when PE disappears after delivery, the cardiac function in patients with PPCM starts to improve as well. However, the majority of cases of PPCM occur in patients who do not develop HDP. Similarly, it is observed that PPCM patients with HDP recover cardiac function more often than those without HDP. Many authors have made different suggestions to explain this. Some have cited the provision of better care to patients with HDP, and others have pointed to an underlying susceptibility in patients without HDP to a worse outcome. What exactly causes this predisposition in patients without HDP who account for most cases of HDP? Seeking an answer to this question may require an immense amount of work in terms of genome sequencing, designing and carrying out clinical trials looking at both etiology and treatment, and establishing registries in different parts of the world. Doing so will not only be a great service to humanity, but will also save valuable resources that are used in the care of these patients.
